# Genetics and Evolution: An iOS Application to Supplement Introductory Courses in Transmission and Evolutionary Genetics

**DOI:** 10.1534/g3.114.010215

**Published:** 2014-04-11

**Authors:** Russell B. Myers, Brandon Millman, Mohamed A. F. Noor

**Affiliations:** * NorthgateArinso Human Resources, South Melbourne, VIC, 3205 Australia; † Pratt School of Engineering, Duke University, Durham, North Carolina 27708; ‡ Biology Department, Duke University, Durham, North Carolina 27708

**Keywords:** app, education, terminology, simulations

## Abstract

Students in college courses struggle to understand many concepts fundamental to transmission and evolutionary genetics, including multilocus inheritance, recombination, Hardy-Weinberg, and genetic drift. These students consistently ask for more demonstrations and more practice problems. With this demand in mind, the “Genetics and Evolution” app was designed to help students (and their instructors) by providing a suite of tools granting them the ability to: (1) simulate genetic crosses with varying numbers of genes and patterns of inheritance, (2) simulate allele frequency changes under natural selection and/ or genetic drift, (3) quiz themselves to reinforce terminology (customizable by any instructor for their whole classroom), *4) solve various problems (recombination fractions, Hardy-Weinberg, heritability, population growth), and (5) generate literally an infinite number of practice problems in all of these areas to try on their own. Although some of these functions are available elsewhere, the alternatives do not have the ability to instantly generate new practice problems or achieve these diverse functions in devices that students carry in their pockets every day.

College students often struggle in genetics more than other introductory biology courses because of its mathematical and probabilistic nature. Although colleges often have mathematics requirements associated with biology-related majors (typically calculus), many students are uncomfortable with applying basic concepts of probability to study genetics (Colon-Berlingeri and Burrowes 2011). Students readily learn the factual material, but they require additional practice and demonstration of mathematical and statistical principles associated with inheritance and population genetics. This discomfort can be potentially alleviated with additional modes of instruction and practice, possibly associated with computer programs (Corbett *et al.* 2010; Sved 2010). Furthermore, the worldwide college population is trending toward greater use of mobile devices rather than desktop and laptop computers, so genetics practice and help provided through such platforms are potentially especially useful.

With these challenges for genetics educators in mind, we present an ad-free iOS app that has resources for both students and faculty, and we share both the app and the underlying code freely. The app provides demonstrations of Mendelian inheritance and population genetics, it assists with calculations common in genetics classes associated with these principles (*e.g.*, recombination fraction), and it generates unique practice problems with which students can practice or professors can design assessments. Furthermore, one part of the app is easily customizable for individual classes, even for faculty without detailed computer programming skills.

## Transmission genetics—Mendelian inheritance

**Figure 1:**
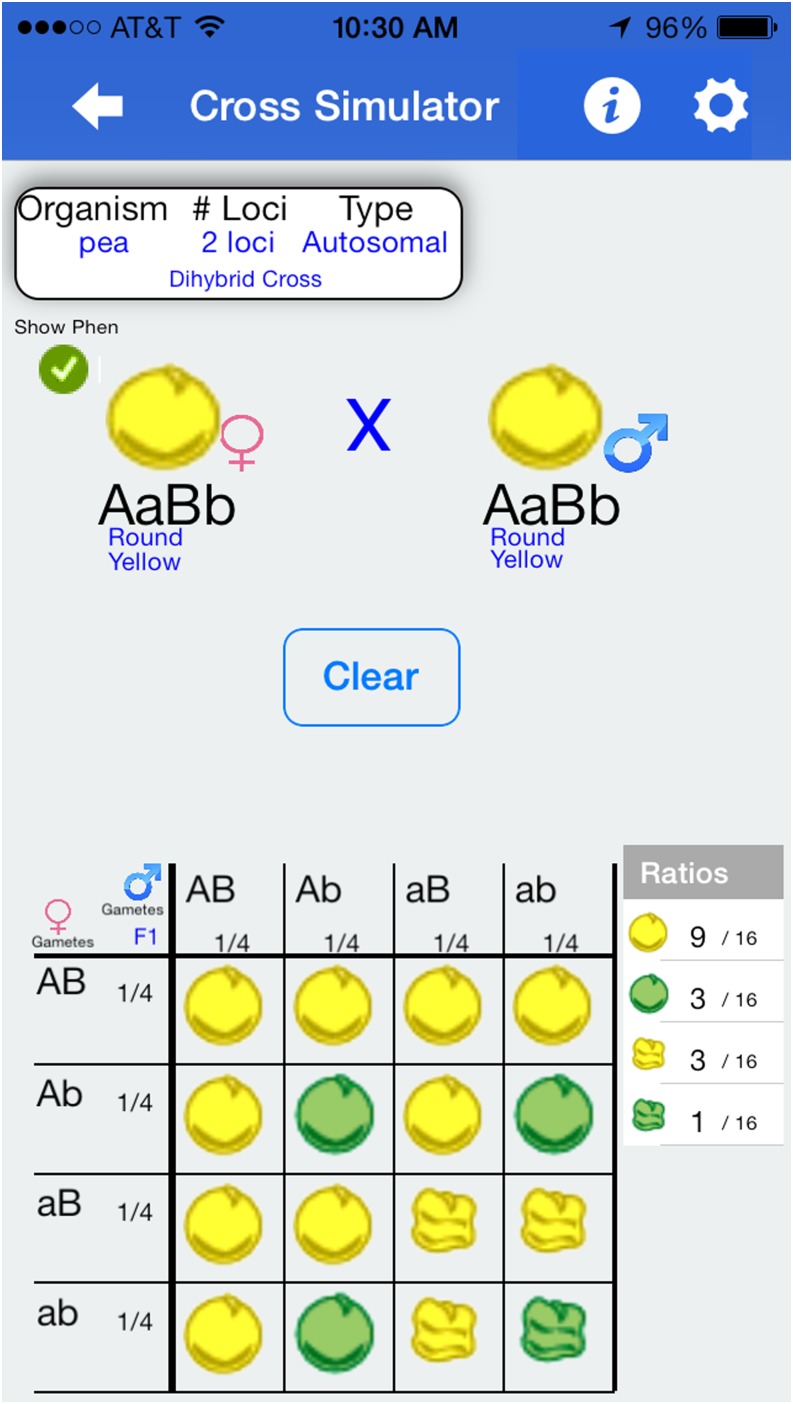
Example of “cross simulator” feature showing products from a dihybrid cross. In this case, the “Show Phenotypes” option is selected, so offspring phenotypes are depicted rather than genotypes both individually and in the overall ratios presented on the lower right side.

Although basic transmission genetics is typically taught in grade school, many incoming university students still struggle with concepts as basic as Mendelian inheritance and dominance (Allchin 2000). Our app provides demonstrations that instructors can use to assist with student understanding. First, with respect to basic inheritance, we provide a “Cross Simulator,” wherein a “male” and “female” of particular genotype and phenotype are crossed (Figure 1). The simulator shows the gametes that go into meiosis as well as the resulting offspring in proportions (both in picture of phenotypes and in numeric ratios). By clicking a button, students can switch between whether they are presented the genotype or phenotype for the offspring (both are shown for parents). Students can go on to select one or more individuals from among the offspring and drag them to the top to become parents for a later generation. The cross is customizable by the user in several respects: the number of (independently assorting) genes affecting the phenotype, whether the genes are X-linked or autosomal, the cross being performed initially, and the fictional organism used. In the present version of the app, all genes are biallelic with complete dominance of one allele, but we are exploring relaxing this assumption.

The cross simulator is designed for student self-exploration—the app defaults to depicting a single-gene cross between two heterozygous individuals, but students can explore more complicated scenarios either on their own or following instructions associated with a course activity. We envision activities wherein students are assigned to explore genotypic and phenotypic proportions from various crosses, as well as general predictive questions of, “Are any grandchildren with X genotype/phenotype possible from a cross between any of the offspring depicted?”

## Transmission genetics—Genetic cross mapping

Many students struggle analyzing and interpreting genetic cross mapping data, as typically presented in the context of Drosophila mutations. They understand that recombination occurs, but moving from genotypes of offspring to inferring phased parental combinations, order of genes, and recombination fractions (map distances) challenges them. To this end, the app provides both a “Problem Solver,” wherein students can check answers to practice problems they were given, and a “Problem Generator,” which randomly generates both practice problems and their solutions. The generated problems can be adjusted for number of offspring or for frequency with which unlinked genes are included (the latter within in the Settings app). At Duke University, instructors of the introductory genetics course use the Problem Generator feature to create exercises implemented in assessments—a professor can generate a list of any number of random numeric recombination fraction problems and solutions (we often use 100) and then select among them for which to deliver to students in proctored tests.

## Population genetics

The app has numerous features associated with teaching population genetic principles. At the most basic level, the Problem Solver and Problem Generator features will work with tests for fit of population genotype numbers to expectations under Hardy-Weinberg assumptions. It also calculates the inbreeding coefficient (F; see Wright 1922) associated with observed deviations from Hardy-Weinberg.

**Figure 2:**
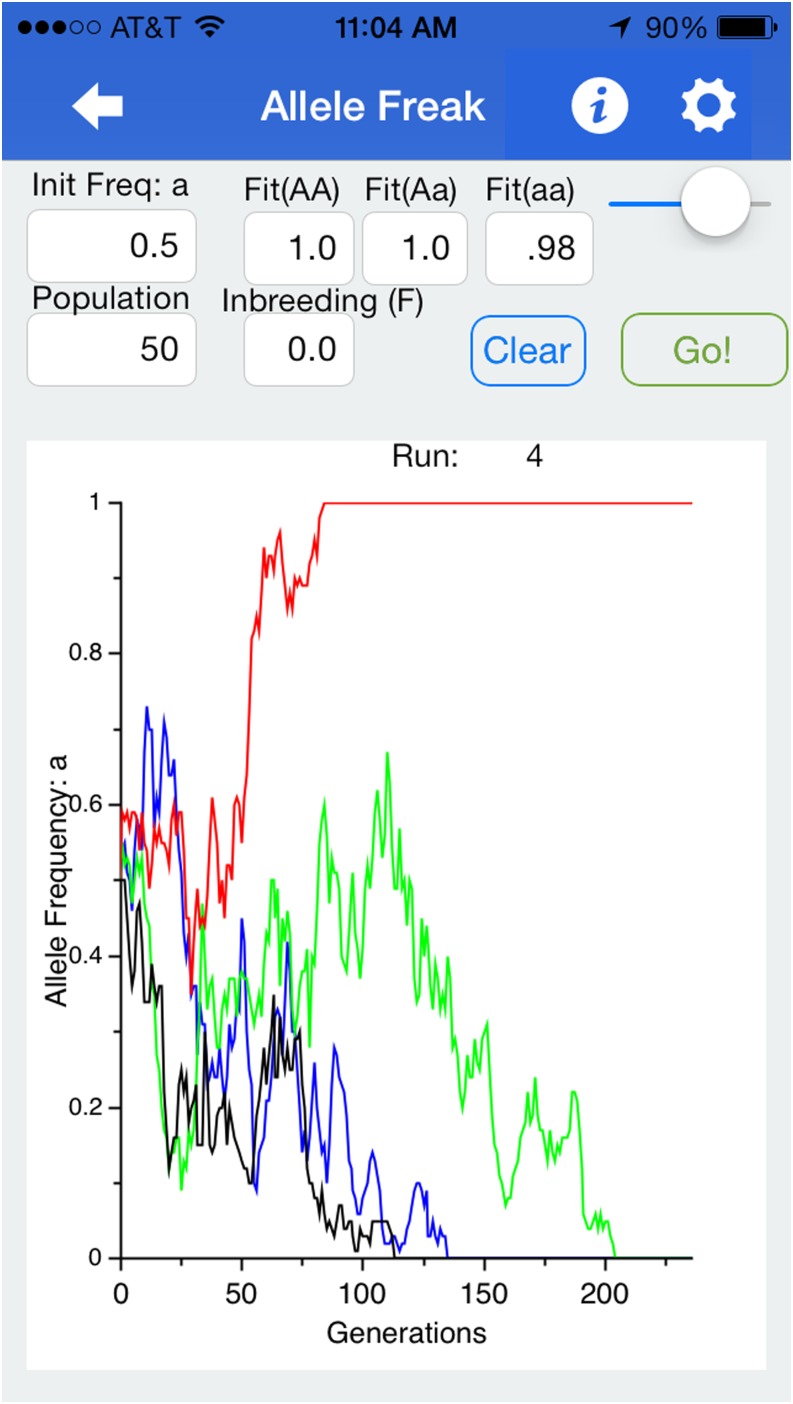
Example of the “Allele Freak” feature depicting allele frequency changes (of “a”) across 250 generations in four populations of size 50, in all of which the genotype “aa” has a slight fitness deficit. This particular example demonstrates how strong genetic drift can sometimes overpower weak natural selection: an overall trend is apparent, but there are outliers.

However, we anticipate students will make greater use of the app’s graphical depictions of evolutionary changes in allele frequency. Within “Allele Freak” (name is a pun on “allele frequency”), students can specify fitnesses associated with all three genotypes at a biallelic locus. They also specify the initial frequency of one allele, the population size (infinity is the default, to demonstrate purely deterministic evolution), and the inbreeding coefficient. The app will then simulate evolutionary change in allele frequency over 400 or more generations. We find this feature especially handy for demonstrating the stochastic effects of genetic drift, including for example that weakly disadvantageous alleles sometimes still spread to fixation (frequency 100%) within a finite population (see example in Figure 2). Table 1 presents several lessons/learning outcomes that can be facilitated by this simulator. The interface features “pinch-to-zoom,” so students can view specific results. The simulation itself works by calculating expected proportions of each genotype and then assigning the number of offspring to genotypes randomly based on the expected proportions. This simulation thus generates each individual each generation, so setting it for very large (but not deterministic/ infinite) populations, or for very long numbers of generations, will slow the run-speed of the app.

**Table 1:** Sample activities/ learning outcomes facilitated with “Allele Freak” feature

• Contrast spread of dominant *vs.* recessive advantageous mutations (or loss of dominant *vs.* recessive detrimental mutations)
• Calculate or verify predicted equilibrium allele frequencies under heterozygote advantage and heterozygote disadvantage
• Contrast effects of genetic drift with varying population sizes
• Identify likelihood of “eventual” fixation of allele via genetic drift and observe that it is predicted by starting allele frequency
• Explore spread/loss of rare beneficial allele in finite population, varying dominance
• Explore effect of inbreeding on speed of loss of detrimental recessive mutation

## Genetics definitions

The app also features a “self-test quiz,” wherein the user is given a definition and asked to pick which of four terms it fits. Although not mathematical or probabilistic, students often have trouble remembering the genetics “jargon,” and this feature was meant to give students the opportunity to practice while waiting for class to begin, on the bus, in line at the store, etc. Importantly, the definitions and terms are customizable. Currently, the app points to a website that we maintain to obtain the definitions initially as well as updates when pushed. However, using the Settings app (labeled “Quiz URL directory”), an instructor can ask their students to point their devices to download a distinct set of definitions and terms generated in-house.

## Synopsis

The Genetics and Evolution app is a work-in-progress, and suggested amendments or features are welcome. In addition to the aforementioned features, the app also solves and generates other types of problems that may arise up in genetics courses, such as calculations of broad-sense heritability using the classic Breeder’s equation and exponential population growth (using either intrinsic rate of increase or birth/ death rates). Overall, we believe that this student-centered app works well to reinforce concepts used in general genetics courses, including those identified in formal inventories designed for teaching and gauging understanding of genetics (Smith *et al.* 2008; Smith and Knight 2012). The underlying code is available as Supporting Information, File S1 and may be adapted for other uses. The app itself is available from the iTunes app store under the name “Genetics and Evolution” at: https://itunes.apple.com/us/app/genetics-and-evolution/id650401749

## Supplementary Material

Supporting Information
