# Supplementary material for: Genetics and Evolution: An iOS Application to Supplement Introductory Courses in Transmission and Evolutionary Genetics
Source: G3 (Bethesda). 2014 Apr 11;4(5):779–81. doi: 10.1534/g3.114.010215 (PMC4025476; doi:10.1534/g3.114.010215)
Supplement: Supporting Information [file supp_4_5_779__index.html]

Genetics and Evolution: An iOS Application To Supplement Introductory Courses in Transmission and Evolutionary Genetics — Genetics and Evolution: An iOS Application to Supplement Introductory Courses in Transmission and Evolutionary Genetics — Supporting Information 

# Genetics and Evolution: An iOS Application to Supplement Introductory Courses in Transmission and Evolutionary Genetics

## Supporting Information for Myers, Millman, and Noor, 2014

**Files in this Data Supplement:**

- File S1 - Source code for the app (.zip, 122 KB)
