# Supplementary material for: Genetics and Evolution: An iOS Application to Supplement Introductory Courses in Transmission and Evolutionary Genetics
Source: G3 (Bethesda). 2014 Apr 11;4(5):779–81. doi: 10.1534/g3.114.010215 (PMC4025476; doi:10.1534/g3.114.010215)
Supplement: Supporting Information [file supp_g3.114.010215_FileS1.zip › _readme.rtf]

Genetics and Evolution (GenEvol)Current version 3.0Author original version: Brandon MillmanAuthor versions 1.1->3.0 Russell B MyersExternal frameworks used:CorePlotDTCoreTextDTFoundationFontasticIconsGHMarkdownParserGRMustacheJRSwizzleMJPopupViewControllerPrettyKitQuickDialogTTOpenInAppActivityUnderscore.mWCAlertViewYRDropdownViewQueries: flipside@netspace.net.au
